# The MDM2 small-molecule inhibitor RG7388 leads to potent tumor inhibition in p53 wild-type neuroblastoma

**DOI:** 10.1038/cddiscovery.2015.26

**Published:** 2015-08-24

**Authors:** A Lakoma, E Barbieri, S Agarwal, J Jackson, Z Chen, Y Kim, M McVay, J M Shohet, E S Kim

**Affiliations:** 1 Division of Pediatric Surgery, Michael E. DeBakey Department of Surgery, Baylor College of Medicine, Houston, TX 77030, USA; 2 Department of Pediatrics, Section of Hematology-Oncology, Texas Children’s Cancer Center, Center for Cell and Gene Therapy, Baylor College of Medicine, Houston, TX 77030, USA; 3 Division of Pediatric Surgery, Department of Surgery, Keck School of Medicine, University of Southern California, Los Angeles, CA 90027, USA

## Abstract

Neuroblastoma is an aggressive pediatric malignancy which is >98% p53 wild-type at diagnosis. As a primary repressor of p53 activity and part of a p53-activated negative feedback loop, targeting of mouse double minute 2 homolog (MDM2) is an attractive therapeutic approach to reactivation of p53. Since development of the first selective MDM2 inhibitor, Nutlin-3a, newer compounds have been developed for increased potency and improved bioavailability. Herein, we sought to determine the efficacy and specificity of a second-generation MDM2 inhibitor, RG7388, in neuroblastoma cell lines and xenografts and examine its effect on the p53-independent pathway of hypoxia-inducible factor-1 alpha (HIF-1*α*)/vascular endothelial growth factor (VEGF). Cell viability and apoptosis studies were performed on the neuroblastoma cell lines, NGP, SH-SY5Y, LAN-5, LAN-5 si-p53 (p53 silenced), and SK-N-AS (p53 null). RG7388 potently decreased cell proliferation and activated p53-dependent apoptosis. Tumor-bearing mice treated with RG7388 demonstrated significant tumor inhibition by 59% in NGP (*P*=0.003), 67% in SH-SY5Y (*P*=0.006), and 75% in LAN-5 (*P*=0.0019) p53 wild-type xenograft tumors, but no inhibitory effect on LAN-5 si-p53 or SK-N-AS p53-silenced/null xenograft tumors. Moreover, RG7388 was found to inhibit the p53-independent pathway of HIF-1*α*/VEGF with decreased gene expression and alteration of angiogenesis. Our study supports the further evaluation of RG7388 as a novel treatment option in p53 wild-type neuroblastoma at diagnosis and relapse.

## Introduction

Neuroblastoma is an aggressive pediatric malignancy arising from the neural ectoderm and represents the most common extra-cranial solid tumor in children. It accounts for 13% of all pediatric cancer mortalities, and despite current regimens of dose-intensive chemotherapy, children who present with metastatic high-risk disease have less than 50% overall survival.^1,2^ Current therapies for neuroblastoma are highly toxic and associated with morbidity and an increased risk of secondary malignancies.^3,4^ As such, targeted therapies with less morbidity and toxicity are actively being sought.

An ideal therapy would be non-genotoxic and manipulate the cancer cell’s innate machinery to obliterate tumor growth, such as activating a latent apoptosis pathway.

P53 is a well-known tumor suppressor and critical regulator of cellular pathways that oppose malignant transformation by inducing cell apoptosis. In neuroblastoma, >98% of new diagnoses have an intact, wild-type p53 with functional activity.^5,6^ Furthermore, the majority of relapsed tumors retain intact downstream p53-dependent apoptotic capacity. The primary negative regulator of p53 is mouse double minute 2 homolog (MDM2), an E3 ubiquitin ligase responsible for the primary ubiquitination of p53. MDM2 is found to be overexpressed in neuroblastoma and other malignancies.^7,8^ In 2004, the first selective and potent MDM2 inhibitors, termed the nutlins, were reported.^9^ Since then, initial clinical and preclinical studies with MDM2 inhibitors have demonstrated promising efficacy of this class of drugs in a number of p53 wild-type adult and pediatric cancers as a single agent or in combination with other targeted therapies.^10–20^ However, many of the initially designed molecules have not successfully been utilized in clinical trials because of limited *in vivo* potency and poor bioavailability.^21,22^ With this in mind, a new generation of MDM2 inhibitors has been developed to optimize potency and bioavailability.^23,24^

The latest generation MDM2 inhibitor RG7388 has demonstrated selective and potent p53 to MDM2 inhibition with improved bioavailability.^24^ Herein, we utilize RG7388 and examine the effect of this novel inhibitor in neuroblastoma both *in vitro* and *in vivo*. Similar to our previous studies in neuroblastoma utilizing the MDM2 inhibitor Nutlin-3a, RG7388 was noted to effectively rescue p53 and activate downstream apoptotic pathways in p53 wild-type cell lines *in vitro*.^20,25^ Using an orthotopic murine model of neuroblastoma, we also demonstrate that RG7388 potently inhibits neuroblastoma tumor growth in p53 wild-type xenografts, and that this inhibitory effect is abrogated in p53 mutated/null tumors *in vivo*. Previously, it has been demonstrated that an important p53-independent pathway by which MDM2 inhibition with Nutlin-3a leads to suppression of hypoxia-inducible factor-1 alpha (HIF-1*α*) and downstream vascular endothelial growth factor (VEGF) expression.^20^ In the current study, we observed that RG7388 leads to inhibition of the HIF-1*α*/VEGF pathway with subsequent alteration and inhibition of angiogenesis. Our data support the efficacy and use of MDM2 inhibitors in p53 wild-type neuroblastoma as a non-genotoxic approach to treating this aggressive malignancy.

## Results

### RG7388 leads to increased expression of p53 and activation of downstream transcription target genes

**Figure 1:**
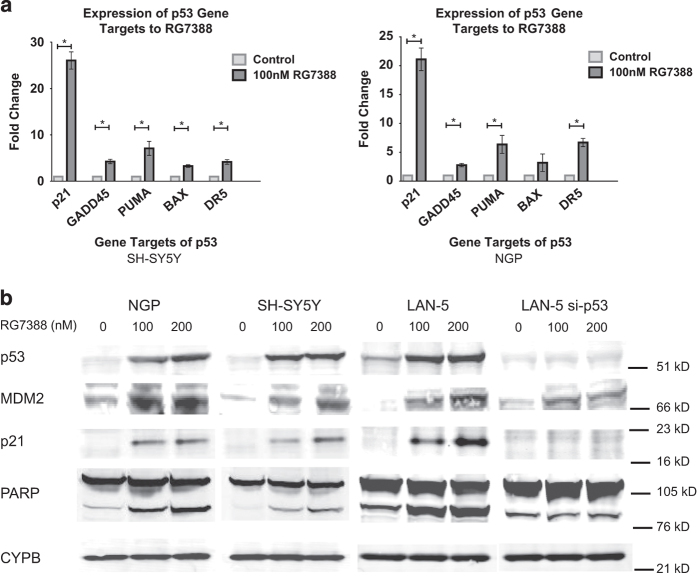
RG7388 upregulates mRNA and protein expression of the p53 pathway. (**a**) MDM2 inhibition with RG7388 increases mRNA expression of p53 transcription targets related to apoptosis in SH-SY5Y (left) and NGP (right) cells. Significant increases with RG7388 treatment is seen for p21, GADD45 (Growth Arrest and DNA Damage genes), PUMA (p53 upregulated modulator of apoptosis), BAX (Bcl-2-associated X protein), and DR5 (death receptor 5) expression in both cell lines by quantitative PCR (*P<0.05). (**b**) Western blot analysis to examine the effect of increasing doses of RG7388 on the human neuroblastoma cell lines NGP, SH-SY5Y, and LAN-5, and LAN-5 si-p53. RG7388 leads to increased p53 protein expression, a compensatory increase in MDM2 expression, and activates p53-mediated apoptosis with an increase in p21 and PARP expression, but not in p53-silenced neuroblastoma cells (LAN-5 si-p53). Numbers on right are approximate molecular weight in kDa.

Inhibition of MDM2 binding to p53 blocks ubiquitin-mediated degradation of p53, leading to accumulation of both cytoplasmic and nuclear p53, with subsequent activation of downstream transcriptional targets such as p21 and induction of apoptotic stress responses.^20,25^ In our current study, we utilized a second-generation MDM2 small-molecule inhibitor, RG7388, which has been found to be more potent and have greater bioavailability than previous generation MDM2 inhibitors.^24^ Utilizing two p53 wild-type cell lines of neuroblastoma (NGP and SH-SY5Y), we assessed gene expression of p53 transcriptional targets in RG7388-treated and untreated neuroblastoma cells. We found that RG7388 significantly upregulates the mRNA expression of p53 transcriptional targets involved in cell arrest (p21 and GADD45), the intrinsic pathway of apoptosis (PUMA and BAX), and the extrinsic pathway of apoptosis (death receptor 5 (DR5)). RG7388 leads to a 26-fold increase in p21 (*P*=0.0018), a 4-fold increase in GADD45 (*P*=0.0072), a 7-fold increase in PUMA (*P*=0.02), a 3-fold increase in BAX (*P*=0.0051), and a 4-fold increase in DR5 (*P*=0.0078) in the SH-SY5Y cell line (Figure 1a, left panel). A similar upregulation of the same genes is observed after RG7388 treatment in the NGP cell line as well (Figure 1a, right panel).

We then assessed the protein expression of p53 as well as p53 downstream targets in neuroblastoma cell lines by western blot analysis. Increasing concentrations of RG7388 (100 and 200 nM) lead to a dose-dependent increase of p53 in the neuroblastoma cell lines NGP (3.6- and 4.4-fold, respectively), SH-SY5Y (8.9- and 10.2-fold), and LAN-5 (4- and 5-fold). However, in the LAN-5 cell line with short-hairpin RNA-p53 silencing (LAN-5 si-p53), the protein expression of p53 was not affected by RG7388 treatment (Figure 1b).

We also evaluated protein expression of the p53 downstream target p21 as well as levels of MDM2, the primary negative regulator of p53. RG7388 (100 and 200 nM) induces increased protein expression of p21 in NGP (6.3- and 7.0-fold), SH-SY5Y (4- and 7.5-fold), and LAN-5 (9.8- and 18.1-fold) cell lines (Figure 1b). Inhibition of MDM2 with RG7388 leads to a rebound increase in expression of MDM2 in NGP (7.9- and 8.2-fold), SH-SY5Y (2.8- and 4.7-fold), and LAN-5 (5.8- and 7.4-fold) cell lines (Figure 1b). However, protein expression of p21 and MDM2 is minimally affected in the LAN-5 si-p53 cell line after RG7388 treatment.

Next, we assessed for evidence of apoptosis by measuring PARP cleavage expression, and demonstrated that increasing concentrations of RG7388 (100 and 200 nM) lead to a dose-dependent increase in apoptosis in NGP (3.5- and 9.8-fold), SH-SY5Y (4- and 6.4-fold), and LAN-5 (3- and 3.8-fold) cell lines (Figure 1b). However, an increase in PARP cleavage is not altered in the LAN-5 si-p53 cell line treated with RG7388. Therefore, consistent with the previous generation of nutlin MDM2 inhibitors, we conclude that RG7388 rescues p53 from ubiquitination and degradation and leads to the activation of downstream p53-dependent apoptosis in neuroblastoma cells.

### RG7388 leads to a p53-dependent decrease in cell proliferation and increase in apoptosis in neuroblastoma

**Figure 2:**
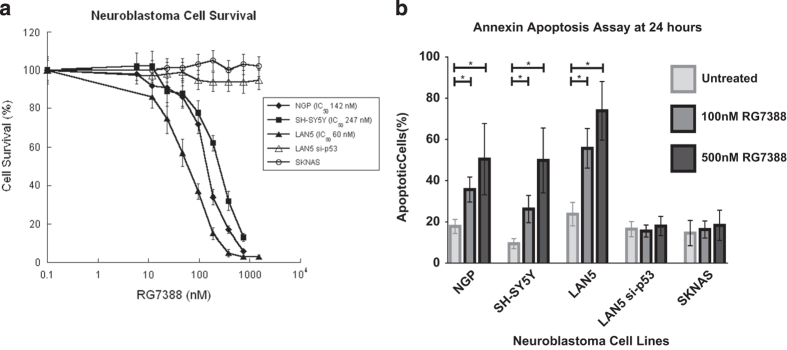
Effect of RG7388 on p53 wild-type and p53 silenced/null neuroblastoma cells *in vitro*. (**a**) RG7388 markedly decreases cell proliferation by MTS assay in NGP, SH-SY5Y, and LAN-5 cells (p53 wild-type) but not in p53 silenced/null cell lines (LAN-5 si-p53 and SK-N-AS). (**b**) RG7388 leads to a significant dose-dependent increase in apoptosis by Annexin apoptosis assay in NGP, SH-SY5Y, and LAN-5 cells but not in LAN-5 si-p53 or SK-N-AS cells (*P<0.05).

To determine the effects of RG7388 on neuroblastoma cells, we treated five neuroblastoma cell lines with RG7388: three cell lines had wild-type p53 (NGP, SH-SY5Y, and LAN-5) and two cell lines with silenced or null-mutated p53 (LAN-5 si-p53 and SK-N-AS). Using a MTS cell proliferation assay, we found that RG7388 treatment leads to a robust decrease in cell proliferation in the three neuroblastoma p53 wild-type cell lines (IC_50_: 142 nM NGP, 247 nM SH-SY5Y, and 60 nM LAN-5; Figure 2a). Treatment with RG7388 had minimal effect on the LAN-5 si-p53 cell line and SK-N-AS cell line (Figure 2a).

We then evaluated apoptosis after RG7388 treatment using an Annexin V assay. Increasing concentrations of RG7388 lead to a dose-dependent increase in apoptosis in p53-wild-type neuroblastoma cell lines (Figure 2b). After RG7388 treatment (100 and 500 nM), there is a 2- and 2.8-fold increase in apoptosis in NGP (*P*=0.011, *P*=0.033), a 2.8- and 5.3-fold increase in SH-SY5Y (*P*=0.014, *P*=0.012), and a 2.3- and 3.1-fold increase in LAN-5 cells (*P*=0.008, *P*=0.005) compared with no treatment. No significant difference in apoptosis was observed in the LAN-5 si-p53 or the p53 mutant SK-N-AS cell line after RG7388 treatment (Figure 2b). These data confirm RG7388 has a potent apoptotic effect on neuroblastoma cells, which is dependent on the presence of wild-type p53.

### RG7388 inhibition of neuroblastoma tumor growth is p53 dependent

**Figure 3:**
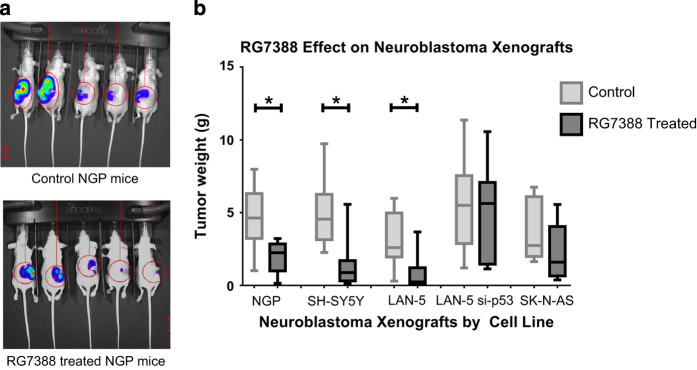
RG7388 inhibits tumor growth in established p53 wild-type neuroblastoma xenografts. (**a**) Representative bioluminescent images of tumor-bearing mice demonstrates tumor inhibition in RG7388-treated mice (bottom) compared with control (top). (**b**) RG7388 treatment significantly inhibits tumor growth of p53 wild-type neuroblastoma xenografts from NGP, SH-SY5Y, and LAN-5 cell lines, but have little effect on xenografts derived from LAN-5 si-p53 and SK-N-AS cell lines (*P<0.01).

To test the effect of RG7388 on tumor growth, we utilized an orthotopic murine model of neuroblastoma. Five cell lines of neuroblastoma (NGP, SH-SY5Y, LAN-5, LAN-5 si-p53, and SK-N-AS) were orthotopically implanted into 100 nude mice (20 mice for each cell line). Three weeks after tumor implantation and verifying tumor engraftment by bioluminescent imaging, mice within each cell line were randomized and treated with either control vehicle (10 mice) or RG7388 (10 mice) daily for 2 weeks. Tumors were followed weekly by bioluminescent imaging, and all mice were killed at 5 weeks post tumor implantation (Figure 3a).

All of the mice tolerated RG7388 treatment well without morbidity, toxicity, or weight loss. At the time the mice were killed, we found that RG7388 treatment significantly suppresses p53 wild-type neuroblastoma xenograft tumor growth by 59% in NGP (*P*=0.003), 67% in SH-SY5Y (*P*=0.006), and 75% in LAN-5 (*P*=0.0019) as compared with vehicle-treated mice (Figure 3b). In p53 null and silenced neuroblastoma xenografts, RG7388 had no significant effect on tumor growth in LAN-5 si-p53 xenografts or SK-N-AS xenografts.

### RG7388 treatment leads to p53-dependent tumor apoptosis in neuroblastoma xenografts

**Figure 4:**
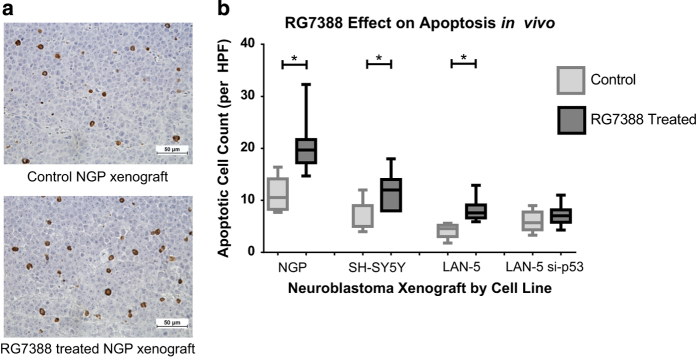
RG7388 treatment induces tumor apoptosis in p53 wild-type neuroblastoma xenografts. (**a**) Representative light microscopy images of caspase-3 immunostaining for apoptosis from NGP xenografts treated with RG7388 and control vehicle (standard bar=50 *μ*m). (**b**) Quantification of caspase-3 immunostaining (positive cells per high-power field) demonstrates a significant increase in apoptotic cells with RG7388 treatment in NGP, SH-SY5Y, and LAN-5 xenograft tumors but not in LAN-5 si-p53 (*P<0.01).

After demonstrating p53-dependent tumor inhibition in neuroblastoma xenografts with RG7388 treatment, RG7388-treated and vehicle-treated neuroblastoma xenografts were assessed for tumor apoptosis by immunohistochemistry for caspase-3 (Figure 4a). In the p53-wild-type neuroblastoma xenografts treated with RG7388, we observed significantly more apoptotic tumor cells per high-power field compared with control vehicle treatment (Figure 4b). In the NGP xenografts, RG7388 leads to a 1.8-fold increase in apoptotic cells compared with control (*P*<0.0001), and in the SH-SY5Y xenograft group, a 1.4-fold increase in apoptotic cells compared with vehicle treatment (*P*=0.0014).

In the LAN-5 xenografts, RG7388 leads to a twofold increase in apoptotic cells compared with control (*P*<0.0001). However, RG7388 did not have a significant effect on tumor apoptosis in the LAN-5 si-p53 xenografts (*P*=0.19; Figure 4b). These data suggest that the primary mechanism by which RG7388 leads to tumor inhibition in p53 wild-type neuroblastoma xenografts is through the activation of p53-mediated tumor apoptosis.

### RG7388 modulates VEGF and angiogenesis in a p53-independent manner

Our previous studies and others have demonstrated that MDM2 activates HIF-1*α* in a p53-independent manner, which leads to downstream vascular endothelial growth factor (VEGF) expression.^20^ Inhibition of MDM2 with the small-molecule inhibitor Nutlin-3a suppresses the HIF-1*α*/VEGF pathway and subsequent VEGF-mediated tumor angiogenesis in neuroblastoma.^20^ In the current study, we sought to determine the effect of RG7388, which belongs to a different class of molecules based on its pyrrolidine ring, on this angiogenesis pathway in neuroblastoma.

**Figure 5:**
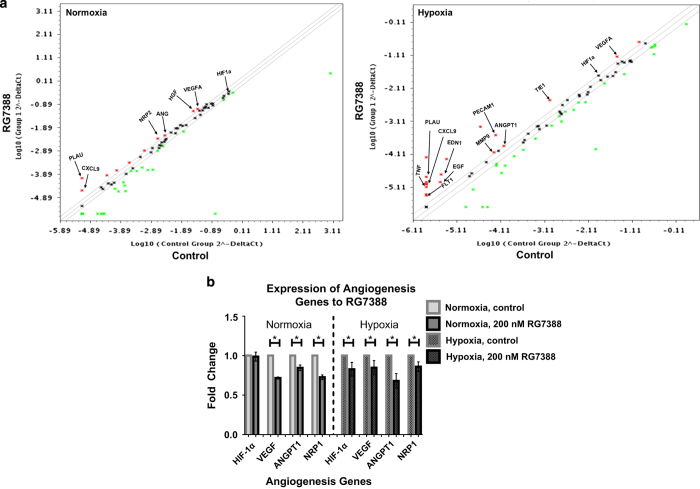
Angiogenesis gene expression analysis with RG7388. (**a**) Scatter plot analysis using a low-density qPCR array for Human Angiogenesis pathway genes in untreated and RG7388-treated (200 nM×6 h) SH-SY5Y cells under normoxic (21% O_2_; left panel) and hypoxic (1% O_2_; right panel) conditions. Compared with normoxic conditions, results show that under hypoxic conditions, RG7388 downregulates the expression of a number of genes associated with angiogenesis, including HIF-1*α*, VEGF, angiopoietin-1 (ANGPT1), and neuropilin-1 (NRP-1). (**b**) Quantitative PCR validation of selected downregulated genes critical to tumor angiogenesis in SH-SY5Y neuroblastoma cells with and without RG7388 under normoxic and hypoxic conditions (*P<0.05).

To determine the effect of RG7388 on the expression of angiogenic genes, a low-density qPCR array for human angiogenesis was performed on the SH-SY5Y neuroblastoma cell line under both normoxic and hypoxic conditions (Figure 5a). Compared with normoxic conditions, there were a number of downregulated genes (red dots) critical to tumor angiogenesis with RG7388 treatment under hypoxic conditions.

These findings were then validated for select individual genes by qPCR in RG7388-treated and untreated SH-SY5Y neuroblastoma cells (Figure 5b). Notably, RG7388 leads to a significant decrease in mRNA expression of the angiogenic factors HIF-1*α*, VEGF, angiopoietin-1 (ANGPT1), and neuropilin-1 (NRP-1, a co-receptor for the VEGF receptor) under both normoxic and hypoxic conditions.

**Figure 6:**
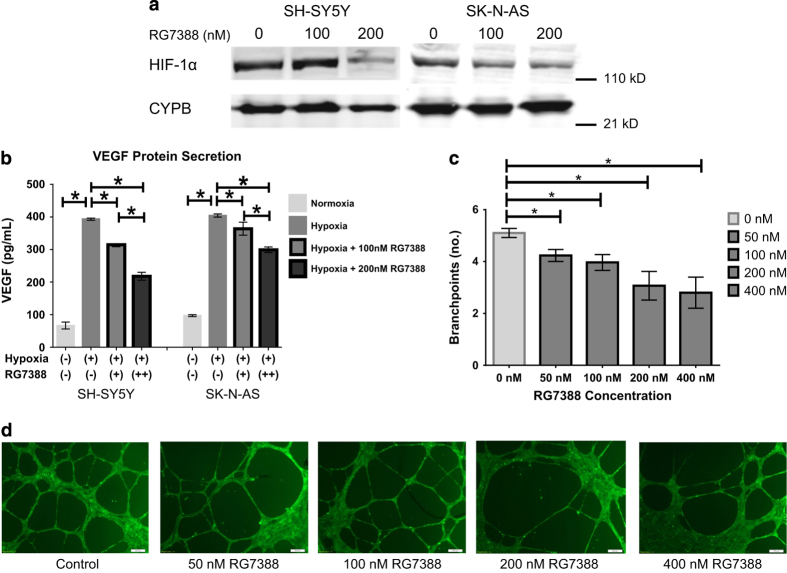
RG7388 inhibits the HIF-1*α*/VEGF pathway and alters angiogenesis. RG7388 treatment of p53 wild-type SH-SY5Y cells and p53 null SK-N-AS cells leads to a decrease in protein expression of (**a**) HIF-1*α* by western blot analysis as well as (**b**) VEGF secretion by ELISA in a dose-dependent manner. (**c**) Using an *in vitro* angiogenesis assay, increasing concentrations of RG7388 leads to a dose-dependent decrease in neovasculature branchpoints, (**d**) as well as a markedly altered, dilated, and distorted vascular phenotype (*P<0.05; standard bar=200 *μ*m).

We then evaluated the protein expression of HIF-1*α* by western blot analysis and VEGF secretion by ELISA in RG7388-treated and untreated neuroblastoma cell lines SH-SY5Y (p53 wild-type) and SK-N-AS (p53 null). Under hypoxic conditions, protein expression of HIF-1*α* decreases by 81% with RG7388 (200 nM) in the SH-SY5Y cell line and decreases by 26 and 49% with RG7388 (100 and 200 nM) in the SK-N-AS cell line (Figure 6a). Further, RG7388 leads to a decrease in VEGF protein secretion in a dose-dependent manner in both the SH-SY5Y cell line (20% at 100 nM, *P*=0.0004, 44.6% at 200 nM, *P*<0.0001) and the SK-N-AS cell line (10% at 100 nM, *P*=0.0266, 26% at 200 nM, *P*<0.0001; Figure 6b). These data suggest that RG7388 suppresses HIF-1*α* and VEGF in a p53*-*independent manner.

We then assessed the effect of RG7388 on endothelial vessel formation utilizing an *in vitro* HUVEC angiogenesis assay. RG7388 significantly altered and decreased angiogenesis with less mesh-like vascular networks with less branchpoints. Untreated HUVEC cells were noted to have 5.1±0.2 branchpoints, whereas RG7388 treatment at various concentrations (50, 100, 200, and 400 nM) leads to a decrease in the number of branchpoints (4.2±0.2, *P*=0.027; 4.0±0.3, *P*=0.015; 3.1±0.6, *P*=0.014; and 2.8±0.6, *P*=0.005; Figure 6c). In addition, we noted that at higher concentrations of RG7388, the vascular networks qualitatively became highly irregular and dilated (Figure 6d).

## Discussion

Remarkably, nearly 100% of *de novo* high-risk neuroblastoma is p53 wild-type with intact downstream apoptotic machinery. In addition, even after multiple rounds of dose-intensive chemotherapy, a large majority of recurrent neuroblastomas maintain p53 functionality.^26,27^ This suggests that for neuroblastoma patients with both *de novo* and relapsed situations, reactivation of p53 and induction of p53-dependent apoptosis may be an effective therapeutic strategy.^26,27^ The oncogene MDM2 is the major negative regulator of p53 and has become the focus of such molecular targeting.^7,8^ MDM2 dramatically shortens the protein half-life of p53 by initiating ubiquitination via its E3 ligase activity. MDM2 inhibitors, which block the specific interaction between MDM2 and p53, can thus generate high levels of intracellular p53 and trigger apoptosis.^9,25^ The first specific and potent small-molecule inhibitor to MDM2, Nutlin-3a, was developed by Vassilev *et al.* in 2004.^9^ Despite potent efficacy *in vitro* and *in vivo* in preclinical studies in multiple p53 wild-type malignancies, Nutlin-3a was found to have poor bioavailability, and as such, new generation MDM2 small-molecule inhibitors were developed with improved potency and pharmacologic properties.^21–24^

In efforts to optimize the Nutlin compounds, Vu *et al.* performed dimethyl substitution of the imidalzoline core and replaced the methoxy group with a *tert*-butyl group to create the first investigational MDM2 inhibitor, RG7112.^23^ Preclinical evaluation of RG7112 demonstrated that the new drug activates the p53 pathway with a potent antitumor effect *in vitro* and *in vivo*.^15,28^ Ray-Coquard *et al.* reported the use of RG7112 in 20 liposarcoma patients, and while many sustained gastrointestinal toxicity, the drug was found to activate the p53 pathway and demonstrate encouraging results.^17^

Shortly after the development of RG7112, Ding *et al.* reported the discovery of RG7388, a more potent and selective p53-MDM2 inhibitor.^24^ This was achieved by altering the stereochemical configuration of the scaffold pyrrolidine, resulting in a compound with similar activation of the p53 pathway as RG7112, but with far more potency and selectivity. Moreover, Ding and colleagues found that RG7388 activates p53 at a significantly reduced concentration than RG7112, and this effect was seen both *in vitro* and *in vivo*.^24^

To date, there has been limited published preclinical data evaluating the efficacy of RG7388. Higgins and colleagues utilized a preclinical model of osteosarcoma in one cell line to study optimal dosing schedules for RG7388 to minimize toxicities associated with daily dosing of RG7112.^29^ They observed that weekly or biweekly dosing of RG7388 is as efficacious as daily dosing yet utilizing lower RG7388 doses. Moreover, results from a phase I clinical study is emerging with RG7388 (also known as RO5503781) in the treatment of advanced, refractory solid tumor malignancies (NCT01462175). Preliminary results show that RG7388 has pharmacodynamic efficacy with increased p53 pathway biomarkers, mild clinical side effects, and evidence of clinical efficacy with stable disease in patients with sarcoma for 17, 22, and 23+ months duration.^30^ In addition, phase I clinical trials are currently evaluating RG7388 as a single agent or in combination with cytarabine in patients with acute myelogenous leukemia (NCT01773408), as well as a trial looking at RG7388 in combination with posaconazole, a CYP3A4 inhibitor, in patients with refractory solid tumor malignancies (NCT01901172).

With promising results from our previous study using Nutlin-3a, we turned our attention to examine the efficacy of the novel inhibitor RG7388 in the pediatric solid tumor, neuroblastoma. Indeed, we confirm that RG7388 significantly upregulates p53 transcriptional targets and p53-mediated apoptosis in p53 wild-type neuroblastoma cell lines and orthotopic xenografts. These results were confirmed to be p53 dependent as knockdown or mutation abrogates the effect. Therefore, the presence of wild-type p53 is critical for the efficacy of RG7388 with subsequent activation of downstream p53-dependent pathways.

MDM2 inhibitors have also been demonstrated to activate broader, p53-independent pathways in cancer as well. The MDM2 inhibitor Serdemetan has been shown to have equivalent potency in both p53 wild-type and p53 mutant acute leukemia cells, the latter by E2F1-mediated apoptosis.^31^ In addition, Nutlin-3a was found to disrupt MDM2–p73 interaction and hence stabilize pro-apoptotic p73 in neuroblastoma, colon carcinoma, and osteosarcoma cells.^32^ Nutlin-3a has also been found to competitively inhibit multi-drug-resistant protein 1 (MDR-1) and sensitize chemotherapy-resistant neuroblastoma and rhabdomyosarcoma cells.^33^ Finally, we previously demonstrated that MDM2 inhibition with Nutlin-3a suppresses HIF-1*α* expression and downstream VEGF expression in neuroblastoma, which correlated with an anti-angiogenic effect in tumor xenografts.^20^

In the current study, similar to Nultin-3a, we observed that RG7388 treatment of neuroblastoma cells leads to a decrease in HIF-1*α* and VEGF mRNA and protein expression. Using an *in vitro* HUVEC angiogenesis assay, we noted that RG7388 leads to altered, dilated vessel morphology with a significant decrease in branchpoints.

In conclusion, we demonstrate that the novel, second-generation MDM2 inhibitor RG7388 potently inhibits tumor growth in neuroblastoma by p53-mediated apoptosis. This tumor inhibitory effect is critically dependent on the presence of wild-type p53, as the effect of RG7388 is attenuated in p53 silenced and p53 null xenografts. Furthermore, we demonstrate that MDM2 inhibition with RG7388 inhibits the p53-independent pathway of HIF-1*α* / VEGF. Considering that nearly all primary neuroblastoma have an intact, wild-type p53 in conjunction with the improved potency and bioavailability of this latest generation MDM2 inhibitor, our data support the use of RG7388 for the treatment of high-risk neuroblastoma and potentially other p53 wild-type solid tumors.

## Materials and Methods

### Cell culture and sources of lines

The human neuroblastoma cell lines SH-SY5Y/luc and NGP/luc were provided by Drs. J Kandel and D Yamashiro (Columbia University, New York, NY, USA); LAN-5 was a gift of Dr. L Metelitsa (Baylor College of Medicine, Houston, TX, USA), and SK-N-AS was obtained from ATCC (American Type Culture Collection, Manassas, VA, USA). All neuroblastoma cell lines were grown at 37 °C in 5% CO_2,_ maintained in RPMI-1640 medium and supplemented with 10% heat-inactivated FBS, 2 mmol/l glutamine, 100 U/ml penicillin, and 100 mg/ml streptomycin. Neuroblastoma cell lines were validated by genotyping and confirmed by the expression of CD56, Nestin, MYCN, and tyrosine hydroxylase within the past 12 months. All cell lines were routinely tested for *Mycoplasma* and Epstein–Barr Virus.

To generate the LAN-5 p53 knockdown cell line (LAN-5 si-p53), we used the lentiviral pLSLP vector containing a short-hairpin RNA sequence targeting p53, a kind gift of Dr. Michael Karin (UCSD, La Jolla, CA, USA). LAN-5 cells were infected for 24 h and following 72 h after transduction, the cells were grown in media containing 1 *μ*g/ml puromycin to select a stably transduced cell line.

Pooled human umbilical vein endothelial cells were purchased (Lonza Inc, Allendale, NJ, USA, Cat C2519A) and grown at 37 °C in 5% CO_2,_ maintained in EBM-2 medium and supplemented with EBM-2 SingleQuots: 10 ml FBS, 0.2 ml hydrocortisone, 2 ml hFGF, 0.5 ml VEGF, 0.5 ml IGF-1, 0.5 ml ascorbic acid, 0.5 ml hEGF, 0.5 ml GA-1000, 0.5 ml heparin (Lonza). The cells were used at passages 3–5.

RG7388 and vehicle (hydroxypropylcellulose/Tween-80) were provided by Hoffmann-La Roche, Nutley, NJ, USA.

### Antibodies and western blot analysis

Primary antibodies for western blot analysis are the following: a-HIF-1*α* (Millipore, Billerica, MA, USA, Cat 07-1585), a-p53 (Santa Cruz Biotechnology, Dallas, TX, USA, Cat sc-126), a-p21 (Santa Cruz Biotechnology, Cat sc-397), a-MDM2 (Millipore, Cat MABE340), a-PARP (Cell Signaling Technology, Beverly, MA, USA, Cat 9542), and a-CyPB (Santa Cruz Biotechnology, Cat sc-20361).

Secondary antibodies are the following: a-mouse IgG IRDye800, a-rabbit IgG IRDye800, and a-goat IgG IRDye700 (Rockland Immunochemicals, Limerick, PA, USA, Cat 610-732-124, 605-430-002, 611-132-122, respectively). Fifty-microgram aliquots of protein from neuroblastoma cells, treated with RG7388 (100 nM, 200 nM for 6 h) and untreated, were electrophoresed and transferred. Cyclophilin B was used for loading control in the experiments. The Odyssey Infrared Imaging System (Li-Cor, Lincoln, NE, USA) with the above secondary antibodies were used for detection and densitometry (Odyssey software v3.0, Li-Cor).

### Quantitative real-time PCR

Neuroblastoma cells (NGP and SH-SY5Y cell lines) were either treated with RG7388 (100 nM for 12 h) or untreated. RNA was extracted from neuroblastoma cells from the two groups and prepared using the miRNeasy Mini Kit (Qiagen, Valencia, CA, USA, Cat 217004), and cDNA was prepared with High Capacity cDNA Reverse Transcription Kit (Applied Biosystems, Grand Island, NY, USA, Cat 4368814) according to the manufacturer’s instructions. Quantitative real-time PCR was performed with Maxima SYBR Green Master Mix (Thermo Scientific, Waltham, MA, USA, Cat K0222) on a Step One Plus Real-Time PCR System (Applied Biosystems).

Primers (Sigma-Aldrich, St. Louis, MO, USA) used are the following:

P21 forward 5′-ATGTCCGTCAGAACCCATG-3′

P21 reverse 5′-CAGTGGTGTCTCGGTGAC-3′

14-3-3σ forward 5′-GGCCATGGACATCAGCAAGAA-3′

14-3-3σ reverse 5′-CGAAAGTGGTCTTGGCCAGAG-3′

GADD45 forward 5′-TGCGAGAACGACATCAACAT-3′

GADD45 reverse 5′-TCCCGGCAAAAACAAATAAG-3′

PUMA forward 5′-CCTGGAGGGTCCTGTACAATCT-3′

PUMA reverse 5′-GCACCTAATTGGGCTCCATCT-3′

NOXA forward 5′-AGCTGGAAGTCGAGTGTGCT-3′

NOXA reverse 5′-TCCTGAGCAGAAGAGTTTGGA-3′

BAX forward 5′-AGCAAACTGGTGCTCAAGG-3′

BAX reverse 5′-GCTGAGGCAGGTGAATCG-3′

Bcl-2 forward 5′-TCCGCATCAGGAAGGCTAGA-3′

Bcl-2 reverse 5′-AGGACCAGGCCTCCAAGCT-3′

DR5 forward 5′-TGACTCATCTCAGAAATGTCAATTCTTA-3′

DR5 reverse 5′-GGACACAAGAAGAAAACCTTAATGC-3′

PERP forward 5′-GGCTTCATCATCCTGGTGAT-3′

PERP reverse 5′-ACAGCAGCCAAGGCAAGGAG-3′

FAS forward 5′-GTGCTGGACCTCTTCCTGAA-3′

FAS reverse 5′-CGGATGCCCAGGATGTGT-3′

GAPDH forward 5′-GGTCGTATTGGGCGCCTGGTC-3′

GAPDH reverse 5′-GCCAGCATCGCCCCACTTGA-3′.

### *In vitro* cell proliferation assay

Proliferation was evaluated in NGP, SH-SY5Y, LAN-5, LAN-5 si-p53, and SK-N-AS neuroblastoma cells treated with RG7388 using the CellTiter 96 Aqueous One Solution Cell Proliferation Assay (Promega, Madison, WI, USA, Cat G3582). Briefly, 3000 cells per well were plated into a 96-well microtiter plate and incubated at 37 °C overnight. Next day, the media were replaced with 100 *μ*l of fresh media, untreated or treated with RG7388 (5.86 nM–1.5 *μ*M titration). Following a 72-h incubation, 20 *μ*l of One Solution Reagent (MTS and phenazine ethosulfate) was added to each well, and the plate was further incubated for 4 h in the dark at 37 °C. Optical density was determined using a 96-well plate reader (DTX 800 multimode detector) at a 490-nm absorbance and analyzed with Multimode Analysis Software (Beckman Coulter, Brea, CA, USA).

### *In vitro* apoptosis assay

Cell apoptosis was evaluated in NGP, SH-SY5Y, LAN-5, LAN-5 si-p53, and SK-N-AS neuroblastoma cells treated with RG7388 with the FITC Annexin V Apoptosis Detection Kit (BD Pharmingen, San Jose, CA, USA, Ca 556547) per recommended protocol.

FITC Annexin V expression was measured by flow cytometry conducted on an LSR II 5-laser flow cytometer (BD Biosciences, San Jose, CA, USA) and analyzed with BD FACSDiva v6.1.2 (BD Biosciences).

### Enzyme-linked immunosorbent assay

VEGF-A was measured in neuroblastoma cell culture supernates treated with RG7388 using the human VEGF Quantikine ELISA Kit and recommended protocol (R&D Systems Inc., Minneapolis, MN, USA, Cat DVE00). SH-SY5Y and SK-N-AS neuroblastoma cells were treated with RG7388 (100 nM, 200 nM for 6 h) or untreated in conditions of hypoxia (1% oxygen saturation, 37 °C). Untreated neuroblastoma cells in normoxia were used as a control. Optical density was determined using a 96-well plate reader (DTX 800 multimode detector) at a 450-nm absorbance with 570 nm wavelength subtraction and analyzed with Multimode Analysis Software (Beckman Coulter).

### *In vitro* low-density human angiogenesis PCR array

SH-SY5Y neuroblastoma cells were either treated with RG7388 (200 nM for 6 h) or untreated in conditions of either normoxia or hypoxia (1% oxygen saturation, 37 °C). RNA was extracted from neuroblastoma cells from the four groups and prepared using the miRNeasy Mini Kit (Qiagen, Cat 217004), cDNA was prepared from 1 *μ*g of total RNA with High Capacity cDNA Reverse Transcription Kit (Applied Biosystems, Cat 4368814). The cDNA was diluted (1:10), and qPCR was performed as described previously.^34^

A low-density Human Angiogenesis RT^2^ Profiler PCR Array (SABiosciences, Valencia, CA, USA, Cat PAHS-024Z) was used to assess the expression of 84 human angiogenesis genes of treated versus untreated SH-SY5Y neuroblastoma cells, in both normoxic or hypoxic conditions.

Validation by quantitative real-time PCR was performed with the following primers (Sigma-Aldrich):

HIF-1*α* forward 5′-GCGCGAACGACAAGAAAAAGA-3′

HIF-1*α* reverse 5′-TCCAAATCACCAGCATCCAG-3′

VEGF forward 5′-AGCTTCCTACAGCACAACAAATGT-3′

VEGF reverse 5′-CGCCTCGGCTTGTCACA-3′

Angiopoietin-1 forward 5′-GCAACTGGAGCTGATGGACACA-3′

Angiopoietin-1 reverse 5′-CATCTGCACAGTCTCTAAATGGT-3′

NRP-1 forward 5′-TGCGCCAAAGATGTCAGAGA-3′

NRP-1 reverse 5′-AGGGCCAACATCAGGGAATC-3′.

### *In vitro* angiogenesis assay

Vessel formation was evaluated in HUVEC cells were either treated with RG7388 or untreated using the Millicell *μ-*Angiogenesis Inhibition Assay (Millipore, Cat MMA125). Briefly, HUVEC cells were suspended at a concentration of 4×10^5^ cells/ml in EBM-2 media either treated with RG7388 (50, 100, 200 and 400 nM) or untreated. Next, 50 *μ*l of appropriate HUVEC cell suspension was plated per well on the *μ*-Angiogenesis Slide containing the polymerized ECMatrix Gel and incubated at 37 °C for 6 h. Calcium AM staining (1 *μ*M final concentration) was added to each well for FITC-filtered fluorescent imaging. Branchpoints were manually counted using light and fluorescent microscopy (Olympus IX71, Olympus DP2-BSW version 2.2 imaging software, Olympus, Waltham, MA, USA).

### *In vivo* tumor studies

The *in vivo* studies were approved by the Institutional Animal Care and Use Committee of Baylor College of Medicine (AN-4810). We utilized our previously published orthotopic mouse model of neuroblastoma,^35^ in which an inoculum of 10^6^ neuroblastoma cells were surgically implanted under the renal capsule of 100 female NCr nude mice (NGP *n*=22, SH-SY5Y *n*=18, LAN-5 *n*=20, LAN-5 si-p53 *n*=20, SK-N-AS *n*=20). Two weeks after implantation and confirmation of successful tumor engraftment by bioluminescent imaging (Xenogen IVIS 100 System, Caliper Life Sciences, Hopkinton, MA, USA), mice were randomly divided into a control group, which received vehicle (hydroxypropylcellulose/Tween-80), and a treatment group, which received RG7388. RG7388 treatment was initiated 3 weeks post implantation by intraperitoneal injection once daily for 14 days at a concentration of 25 mg/kg/day for mice implanted with NGP and SH-SY5Y, and 35 mg/kg/day for mice implanted with LAN-5, LAN-5 si-p53, and SK-N-AS. Five weeks post implantation, all mice were killed, and xenograft tumors resected and weighed. Tumors were preserved in 4% paraformaldehyde for immunohistochemical analysis.

### Immunohistochemistry

Immunostaining was performed on 5 *μ*m sections of xenograft tumor tissue preserved in paraffin from mice with NGP and SH-SY5Y xenografts using the following antibodies: a-Caspase-3 (Cell Signaling, Cat 9661) and a-CD31 (BD Pharmingen, Cat 557355).

### Quantification of apoptosis

Cleaved caspase-3-positive NGP and SH-SY5Y neuroblastoma cells were manually counted and averaged from 10 high-power fields per xenograft using light microscopy (Olympus IX71, Olympus DP2-BSW version 2.2 imaging software).

### Digital quantification of vasculature

Quantitative assessment of angiogenesis from all xenografts was performed using Nikon Imaging System- Elements AR Analysis (4.20.01 64-bit software; Nikon, Tokyo, Japan). High-power field digital images from immunostained xenograft tumor for vascular elements (CD31) were analyzed and positive pixels quantified.

### Statistical analysis

Western blot analysis, qPCR, and ELISA were all performed in triplicate, and data were compared using Student’s *t*-test. Tumor weights were expressed as mean±S.E.M.

Data from digital image analysis were expressed as mean±S.E.M. and compared by Student’s *t*-test. Incidence of metastasis was calculated by Fisher’s exact test and metastatic burden was calculated by Kruskal–Wallis method. Tumor weights were compared using the Kruskal–Wallis method. A *P*-value of<0.05 was deemed statistically significant.

## Abbreviations

MDM2: mouse double minute 2 homolog
HIF-1*α*: hypoxia-inducible factor-1 alpha
VEGF: vascular endothelial growth factor
